# Survival advantage of Asian metastatic prostate cancer patients treated with external beam radiotherapy over other races/ethnicities

**DOI:** 10.1007/s00345-021-03720-7

**Published:** 2021-05-12

**Authors:** Christoph Würnschimmel, Mike Wenzel, Claudia Collà Ruvolo, Luigi Nocera, Zhe Tian, Fred Saad, Alberto Briganti, Shahrokh F. Shariat, Philipp Mandel, Felix K. H. Chun, Derya Tilki, Markus Graefen, Pierre I. Karakiewicz

**Affiliations:** 1 Martini-Klinik Prostate Cancer Center, University Hospital Hamburg-Eppendorf, Martinistraße 52, 20246 Hamburg, Germany; 2 Cancer Prognostics and Health Outcomes Unit, Division of Urology, University of Montréal Health Center, Montréal, QC Canada; 3 Department of Urology, University Hospital Frankfurt, Frankfurt am Main, Germany; 4 Department of Neurosciences, Reproductive Sciences and Odontostomatology, University of Naples Federico II, Naples, Italy; 5 Department of Urology and Division of Experimental Oncology, URI, Urological Research Institute, IRCCS San Raffaele Scientific Institute, Milan, Italy; 6 Department of Urology, Comprehensive Cancer Center, Medical University of Vienna, Vienna, Austria; 7 Departments of Urology, Weill Cornell Medical College, New York, NY USA; 8 Department of Urology, University of Texas Southwestern, Dallas, TX USA; 9 Department of Urology, Second Faculty of Medicine, Charles University, Prague, Czech Republic; 10 Institute for Urology and Reproductive Health, I.M. Sechenov First Moscow State Medical University, Moscow, Russia; 11 Division of Urology, Department of Special Surgery, Jordan University Hospital, The University of Jordan, Amman, Jordan; 12 Department of Urology, University Hospital Hamburg-Eppendorf, Hamburg, Germany

**Keywords:** Metastatic prostate cancer, External beam radiotherapy, Cancer-specific mortality, Other-cause mortality, Race/ethnicity

## Abstract

**Purpose:**

To assess the effect of race/ethnicity in cancer-specific mortality (CSM) adjusted for other-cause mortality (OCM) in metastatic prostate cancer patients (mPCa) treated with external beam radiotherapy (EBRT) to the prostate.

**Methods:**

We relied on the Surveillance, Epidemiology, and End Results (SEER) database to identify Caucasian, African-American, Hispanic/Latino and Asian mPCa patients treated by EBRT between 2004 and 2016. Cumulative incidence plots displayed CSM after adjustment for OCM according to race/ethnicity. Propensity score matching accounted for patient age, prostate-specific antigen, clinical T and N stages, Gleason Grade Groups and M1 substages. OCM adjusted multivariable analyses tested for differences in CSM in African-Americans, Hispanic/Latinos and Asians relative to Cauacasians.

**Results:**

After 3:1 propensity score matching and OCM adjustment, Asians exhibited lower CSM at 60 and 120 months (48.2 and 60.0%, respectively) compared to Caucasians (66.7 and 79.4%, respectively, *p* < 0.001). In OCM adjusted multivariable analyses, Asian race/ethnicity was associated with lower CSM (HR 0.66, CI 0.52–0.83, *p* < 0.001). Conversely, African-American and Hispanic/Latino race/ethnicity did not affect CSM. OCM rates were comparable between examined races/ethnicities.

**Conclusion:**

In the setting of mPCa treated with EBRT, Asians exhibit lower CSM than Caucasians, African-Americans and Hispanic/Latinos. This observation may warrant consideration in prognostic stratification schemes for newly diagnosed mPCa patients.

## Background

Survival in metastatic prostate cancer (mPCa), despite promising new systemic therapies, remains low [1]. Due to its heterogeneity at presentation, no “one fits all” treatment strategy for mPCa is available [2]. Data from the multi-arm, multi-stage “STAMPEDE” trial investigating androgen-deprivation therapy (ADT) versus ADT plus external beam radiotherapy (EBRT) to the prostate revealed added survival benefit of EBRT to the prostate in a subgroup of patients with low-volume metastatic burden [3]. Since then, EBRT to the prostate in low-volume mPCa became standard of care and complements the multitude of systemic treatment options that are available [4–6]. Recently, the efficacy of EBRT to the prostate in low-volume mPCa was also validated in North American patients [7].

However, no stratification according to race/ethnicity was applied in neither the original STAMPEDE trial nor in the subsequent North American validation study. Based on evidence suggesting racial/ethnic differences in mPCa survival outcomes, it is of interest to test whether some racial/ethnic groups may benefit of EBRT more than others [8–10]. We addressed this void and tested for survival differences between Caucasian, African-American, Hispanic/Latino and Asian mPCa patients who received EBRT to the prostate within the SEER database. Although SEER does not provide information on applied radiation doses, types or fields, to the best of our knowledge, there is no basis for substantial race/ethnicity-related differences in this regard. For this reason, we relied on the SEER-derived EBRT variable in mPCa patients as surrogate for the type of treatment that was performed within the STAMPEDE trial. We hypothesized, that after adjustment for other-cause mortality (OCM), differences may exist in cancer-specific mortality (CSM) between different racial/ethnic groups.

## Patients and methods

Within the SEER database, we identified newly diagnosed mPCa Caucasian, African-American, Hispanic/Latino and Asian patients who were treated with EBRT to the prostate between 2004 and 2016. These selection criteria resulted in a cohort of 4282 assessable patients: 2737 Caucasians, 797 African-Americans, 469 Hispanic/Latinos and 252 Asians. Patient age at diagnosis, year of diagnosis, prostate-specific antigen (PSA) at diagnosis, clinical T and N stage (cT, cN) as well as biopsy Gleason Grade Group (GGG) and M1 substages (M1a, M1b and M1c according to the seventh Edition of the American Joint Committee on Cancer Staging Manual, AJCC [11]), represented covariates. The outcome variable of interest was CSM. OCM was also quantified and used for adjustment in competing risks analyses focusing on CSM.

### Statistical analyses

The first step of the analyses focused on the overall cohort of 4282 patients. Here, cumulative incidence plots displayed CSM and OCM according to each race/ethnicity*.* Furthermore, univariable and multivariable competing risks regression models tested the effect of race/ethnicity on CSM after adjustment for OCM.

In the second step of the analyses, we relied on three subgroup analyses. Here, we sequentially compared CSM of non-Caucasians to Caucasians. The first subgroup analysis focused on African-Americans (versus Caucasians), the second on Hispanic/Latinos (versus Caucasians) and the third on Asians (versus Caucasians). For all subgroups, 3:1 propensity score matching was applied. Matching variables consisted of age at diagnosis (in one year intervals), PSA at diagnosis (in 5 ng/ml intervals), cT1-4 stages (1:1 ratio), GGG 1–5 (1:1 ratio), cNx/cN0/cN1 (1:1 ratio) stages and M1a-c (1:1 ratio) substages. After matching, cumulative incidence plots displayed CSM for (A) Caucasians vs. African-Americans, (B) Caucasians vs. Hispanic/Latinos and (C) Caucasians vs. Asians. Thereafter, competing risks regression tested for race/ethnicity differences in CSM between each of the three subgroups (A, Caucasians vs. African-Americans, B, Caucasians vs. Hispanic/Latinos, C, Caucasians vs. Asians). In all three subgroup analyses, multivariable adjustment relied on the same covariables as in the overall multivariable analysis [12].

R software environment for statistical computing and graphics (version 3.4.0 for MAC OS X; http://www.r-project.org/) was used for all statistical analyses [13]. Descriptive statistics included frequencies and proportions for categorical variables. Medians and interquartile-ranges (IQR) were reported for continuously coded variables. Chi-square and Log-rank tested the statistical significance in proportions and survival differences. All tests were two-sided with a level of significance set at *p* < 0.05.

## Results

### Study population

African-Americans were youngest (median 63 years), followed by Hispanic/Latinos (median 66 years), Caucasians (median 68 years) and Asians (median 70 years). No differences between races/ethnicities were identified according to local cancer characteristics (cT stage, cN stage, GGG and PSA, all *p* ≥ 0.07), but significant differences according to M1 (a–c) substages at diagnosis were recorded between races/ethnicities (*p* < 0.001, Table 1). In this regard, the most common location of metastases for all races/ethnicities was bone (M1b; 67.7% in Caucasians, 63.6% in African-Americans, 61.5% in Hispanic/Latinos and 62.7% in Asians), followed by visceral metastases (M1c; 16.3% in Caucasians, 19.3% in African-Americans, 22.4% in Hispanic/Latinos and 21.8% in Asians) and lastly distant lymph nodes (M1a, 3.7% in Caucasians, 4.1% in African-Americans, 3.4% in Hispanic/Latinos and 0.8% in Asians).

**Table 1:** Patient characteristics of 4282 metastatic (M1) prostate cancer patients of four racial/ethnic groups (Caucasian, African-American, Hispanic/Latino, Asian) treated with external beam radiotherapy between 2004 and 2016 within the Surveillance, Epidemiology and End Results database

	Overall (*n* = 4282)	Caucasian (*n* = 2737)	African-American (*n* = 797)	Hispanic/Latino (*n* = 496)	Asian (*n* = 252)	*p* value
Age, years (median, IQR)	67 (60–75)	68 (61–77)	63 (57–71)	66 (59–74)	70 (63–76)	< 0.001
*SES (n, %)*						< 0.01
1st quartile	1167 (27.3)	960 (35.1)	127 (15.9)	50 (10.1)	30 (11.9)	
2nd-3rd-4th quartile	3115 (72.7)	1777 (64.9)	670 (84.1)	446 (89.9)	222 (88.1)	
*PSA, ng/ml (n, %)*						0.6
0–9.9	492 (11.5)	353 (12.9)	60 (7.5)	53 (10.7)	26 (10.3)	
10.0–19.9	483 (11.3)	341 (12.5)	64 (8.0)	50 (10.1)	28 (11.1)	
20.0–49.9	648 (15.1)	457 (16.7)	90 (11.3)	57 (11.5)	44 (17.5)	
≥ 50.0	2659 (62.1)	1586 (57.9)	583 (73.1)	336 (67.7)	154 (61.1)	
*Clinical stage*						0.24
cT1-2	2409 (56.3)	1544 (56.4)	455 (57.1)	288 (58.1)	122 (48.4)	
cT3-4	1018 (23.8)	654 (23.9)	187 (23.5)	108 (21.8)	69 (27.4)	
Unknown	855 (20.0)	539 (19.7)	155 (19.4)	100 (20.2)	61 (24.2)	
*GGG (n, %)*						0.8
I	138 (3.2)	84 (3.1)	28 (3.5)	16 (3.2)	10 (4.0)	
II–III	608 (14.2)	377 (13.8)	110 (13.8)	85 (17.1)	36 (14.3)	
IV–V	2524 (58.9)	1628 (59.5)	472 (59.2)	281 (56.7)	143 (56.7)	
Unknown	1012 (23.6)	648 (23.7)	187 (23.5)	114 (23.0)	63 (25.0)	
*Regional LN (n,%)*						0.07
cN0	2275 (53.1)	1476 (53.9)	424 (53.2)	244 (49.2)	131 (52.0)	
cN1	1136 (26.5)	726 (26.5)	221 (27.7)	130 (26.2)	59 (23.4)	
cNX	871 (20.3)	535 (19.5)	152 (19.1)	122 (24.6)	62 (24.6)	
*Metastasis location (n, %)*						< 0.001
Bone	2821 (65.9)	1851 (67.6)	507 (63.6)	305 (61.5)	158 (62.7)	
Distant lymph nodes	152 (3.5)	100 (3.7)	33 (4.1)	17 (3.4)	2 (0.8)	
Visceral	767 (17.9)	447 (16.3)	154 (19.3)	111 (22.4)	55 (21.8)	
Unknown	92 (2.1)	53 (1.9)	29 (3.6)	8 (1.6)	2 (0.8)	
*Chemotherapy (n, %)*						0.08
No/Unknown	3711 (86.7)	2373 (86.7)	681 (85.4)	426 (85.9)	231 (91.7)	
Yes	571 (13.3)	364 (13.3)	116 (14.6)	70 (14.1)	21 (8.3)	

*Cauc* Caucasian, *AA* African-American, *SES* socioeconomic status, *PSA* prostate-specific antigen, *GGG* Gleason Grade Group, *LN* lymph node

### Cancer-specific mortality and other-cause mortality in the overall cohort

**Fig. 1:**
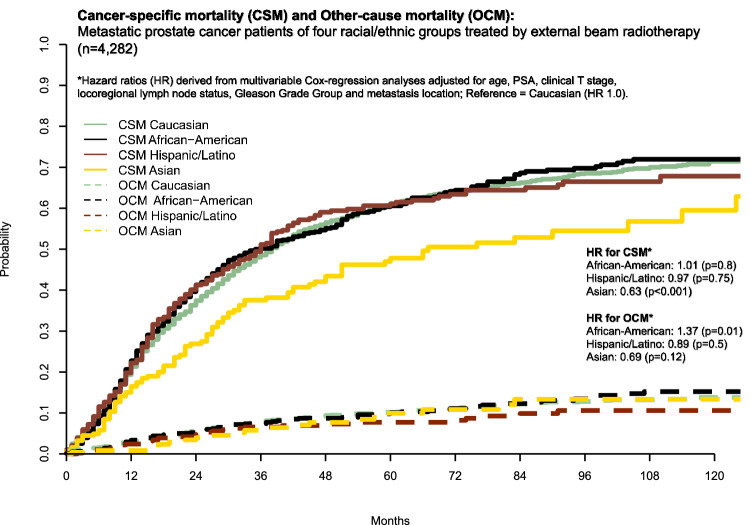
Cumulative incidence plot displaying cancer-specific mortality (CSM) and other-cause mortality (OCM) of 4282 metastatic prostate cancer (mPCA) patients of four racial/ethnic groups, treated by external beam radiotherapy to the prostate between 2004 and 2016 within the Surveillance, Epidemiology and End Results database

In the overall cohort, CSM rates at 60 and 120 months were 61.1% and 71.5% in Caucasians, 60.6% and 72.0% in African-Americans, 61.0% and 67.8% in Hispanic/Latinos and 47.8% and 59.5% in Asians. OCM rates showed minimal differences according to racial/ethnic groups (Fig. 1). In subgroup analyses, that relied on multivariable competing risks regression models, the comparison of African-Americans versus Caucasians resulted in a HR for CSM of 1.01 (CI 0.90–1.13, *p* = 0.8) for African-Americans. In the subgroup comparing Hispanic/Latinos versus Caucasians, the HR for CSM was 0.97 (CI 0.85–1.12, *p* = 0.75) for Hispanic/Latinos. In the subgroup comparing Asians versus Caucasians, HR for CSM was 0.63 (CI 0.51–0.77, *p* < 0.001), favoring Asians.

### Propensity score adjusted competing risks analyses in African-Americans versus Caucasians

**Fig. 2:**
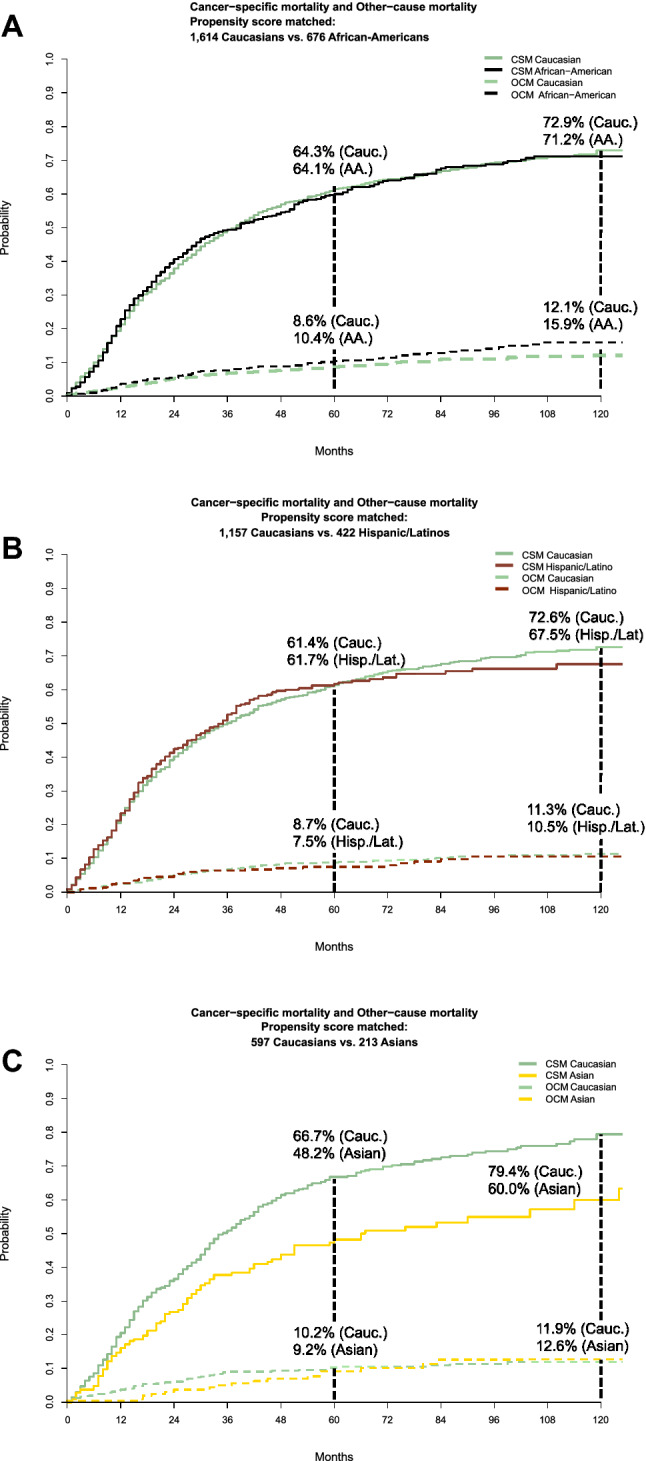
**a–c** Cumulative incidence plot displaying cancer-specific mortality (CSM) and other-cause mortality (OCM) after 3:1 propensity score matching of Caucasian versus African-American patients (**a**), Caucasian versus Hispanic/Latino patients (**b**) and Caucasian versus Asian patients (**c**), treated by external beam radiotherapy to the prostate between 2004 and 2016 within the Surveillance, Epidemiology and End Results database. Propensity score matching was performed according to age at diagnosis (by each year), prostate-specific antigen at diagnosis (by steps of 5 ng/ml), cT1-4, Gleason Grade Groups 1–5, cNx/cN0/cN1 stages and M1a-c substages

After propensity score matching between African-Americans and Caucasians, the cohort consisted of 676 African-Americans versus 1614 Caucasians. Using this matched cohort, cumulative incidence plots revealed CSM of 64.1% for African-Americans and 64.3% for Caucasians at 60 months. CSM at 120 months was 71.2% in African-Americans and 72.9% in Caucasians (Fig. 2a). In multivariable propensity score matched competing risks analyses, adjusted for OCM, a HR of 1.0 (CI 0.88–1.12, *p* = 0.94) for CSM in African-Americans was recorded.

### Propensity score adjusted competing risks analyses in matched Hispanic/Latinos versus Caucasians

After propensity score matching between Hispanic/Latinos and Caucasian, the cohort consisted of 422 Hispanic/Latinos versus 1157 Caucasians. Using this matched cohort, cumulative incidence plots revealed CSM of 61.7% in Hispanic/Latinos and 61.4% in Caucasians at 60 months. CSM at 120 months was 67.5% in Hispanic/Latinos and 72.6% in Caucasians (Fig. 2b). In multivariable propensity score matched competing risks analyses, adjusted for OCM, a HR of 0.96 (CI 0.83–1.13, *p* = 0.64) for CSM in Hispanic/Latinos was recorded.

### Propensity score adjusted competing risks analyses in matched Asians versus Caucasians

After propensity score matching between Asians and Caucasians, the cohort consisted of 213 Asians versus 597 Caucasians. Using this matched cohort, cumulative incidence plots revealed CSM of 48.2% in Asians and 66.7% in Caucasians at 60 months. CSM at 120 months was 60.0% in Asians and 79.4% in Caucasians (Fig. 2c). In multivariable propensity score matched competing risks analyses, adjusted for OCM, a HR of 0.66 (CI 0.52–0.83, *p* < 0.001) for CSM in Asians was recorded.

## Discussion

We hypothesized that important survival differences may exist between race/ethnicity groups in newly diagnosed mPCa treated with EBRT to the prostate. To test our hypothesis, we relied on CSM and OCM data recorded in the SEER database between 2004 and 2016. Our analyses yielded several noteworthy findings.

First, considering patient and tumor characteristics, we generally recorded no meaningful differences between race/ethnicity at initial presentation. Two exceptions were identified. The first one consisted of oldest age at presentation in Asians (median 70 years), followed by Caucasians (median 68 years), Hispanic/Latinos (median 66 years) and African-Americans (median 63 years), in that order. Additionally, Asians also exhibited the second highest rates of visceral metastases (21.8%) after Hispanic/Latinos (22.4%), compared to African-Americans (19.3%) and Caucasians (16.3%). These observations indicate that Asian patients are disadvantaged with respect to survival probability based on more advanced age and based on highest rate of visceral metastasis. In consequence, Asian patients would be expected to exhibit worse survival characteristics than other race/ethnic groups, which was not the case in the current analyses, since Asians exhibited most favorable survival outcomes.

In the second part of the analyses, we focused on CSM. Here, we relied on competing risks regression to account for the confounding effect of OCM. This methodology was applied in univariable, as well as in multivariable analyses. In multivariable analyses, besides adjustment for OCM, our modeling strategy also adjusted for residual differences in patient and prostate cancer characteristics that may still exist between race/ethnic groups despite absence of statistically significant findings identified in descriptive analyses (Table 1). Here, Asians exhibited lower CSM (47.8% at 60 months and 59.5% at 120 months) than Caucasians (61.1% at 60 months and 71.5% at 120 months), Hispanic/Latinos (61.0% at 60 months and 67.8% at 120 months) and African-Americans (60.6% at 60 months and 72.0% at 120 months). Conversely, no statistically significant differences were recorded between African-Americans and Caucasians, as well as between Hispanic/Latinos and Asians. These observations suggest that despite multivariable adjustment for residual differences in patient and prostate cancer characteristics, as well as after adjustment for OCM, Asian mPCa patients treated with EBRT to the prostate exhibit better CSM outcomes than the three other examined racial/ethnic groups.

In the third part of the analysis, we introduced an additional measure for further reduction of confounding, namely propensity score matching. This methodology represents the closest possible approximation of a randomized study design, within a retrospective cohort. However, propensity score matching is only applicable for comparison of no more than two groups. In consequence, we performed three separate subgroup analyses that compared CSM between A: Caucasians versus African-Americans, B: Caucasians versus Hispanic/Latinos and C: Caucasians versus Asians. In each of the comparisons propensity score matching was applied, in addition to competing risks methodology that adjust for OCM as well as in addition to multivariable adjustment. The results of the three subgroup analyses validated the observation that Asian patients exhibit more favorable CSM than Caucasians (HR 0.66, *p* < 0.001). Similarly, subgroup analyses between Caucasians versus African-Americans and Caucasians versus Hispanic/Latinos also validated the lack of statistically significant differences in CSM between those three racial/ethnic groups.

To the best of our knowledge, we are the first to examine racial/ethnic group differences in mPCa treated with EBRT. However, Asian prostate cancer patients have also been found to have more favorable prognosis than other races/ethnicities in other contexts. For example, in a recent report by Deuker et al., lower CSM of Asian patients compared to Caucasian patients in both localized PCa as well as mPCa has been observed [14]. However, in their analyses, Deuker et al. did not focus on EBRT-treated mPCa patients. Furthermore, our findings also contrast with other reports that stated worse survival rates in African-American prostate cancer patients or more favorable survival in Hispanic/Latino prostate cancer patients [15–19]. However, also none of these reports focused on a subgroup of mPCa patients treated by EBRT. In consequence, our results cannot be directly compared to any other previous study. Apart from potentially other unmeasured prognostic factors within the SEER database, it is a matter of discussion whether differences in modifiable factors (e.g. lifestyle and dietary, access to health-care, socioeconomic status) and/or non-modifiable factors (e.g. genetic factors or differences in testosterone levels, radiosensitivity of the prostate, response to systemic treatment), might play a role in the lower CSM of Asian mPCa patients [8, 9].

Taken together, we performed a detailed analysis of the effect of race/ethnicity on CSM in newly diagnosed mPCa treated with EBRT to the prostate. We observed lower CSM in Asians, relative to Caucasians. Conversely, no differences in CSM were recorded between African-Americans and Caucasians as well as Hispanic/Latinos and Caucasians. The observed CSM advantage of Asian patients persisted despite detailed multivariable adjustment, adjustment for OCM as well as propensity score matching for all available patient and prostate cancer characteristics. In consequence, our observations provide robust, albeit retrospective evidence that the prognosis of newly diagnosed Asian mPCa patients treated with EBRT to the prostate is better than that of other racial/ethnic groups. This observation could not have been made in the original STAMPEDE [3] trial, since stratification for race/ethnicity was neither planned nor possible. Our findings should ideally be validated within other large scaled prospective or retrospective databases. Unfortunately, the nature of the SEER database does not allow to test for patient or disease characteristics that could explain why Asian patients displayed better survival.

It is of note that we could not strictly apply the CHAARTED [6] study definition of low-volume newly diagnosed mPCa according to available fields within the SEER database. However, EBRT to the prostate is only guideline-recommended in low-volume mPCa [2]. Nevertheless, no evidence existed to support EBRT to the prostate in patients other than those with low-volume mPCa. In consequence, it is highly unlikely, that a significant proportion of our individuals harbored high volume disease. Moreover, no evidence suggests that clinically meaningful differences in mPCa existed between racial/ethnic groups, as evidenced by absence of significant differences in clinical cancer characteristics such as PSA, GGG or cT/N stages. Despite some minor differences in M1 substage distributions between races/ethnicities, we accounted for these confounders by the means of propensity score matching. In consequence, differential bias in EBRT use due to differences in PCa characteristics between racial/ethnic groups is unlikely operational in our cohort. However, SEER neither accounts for the type or dose of applied systemic treatment nor for the applied radiation dose or type. Nonetheless, there is no basis for differences in rates or types of treatments administered according to racial/ethnic groups. In consequence, it is unlikely that those limitations represented sources of differential bias or confounding.

## Conclusion

In the setting of EBRT-treated mPCa patients, Asian race/ethnicity exhibits more favorable CSM than Caucasian, African-American and Hispanic/Latino race/ethnicity. This observation may warrant consideration in prognostic stratification schemes for newly diagnosed mPCa patients.

## Data Availability

All datasets generated for the study are publicly available. Data will be shared to bona fide researchers.

## Code availability

Used code will be shared to bona fide researchers.
